# Dynamic analysis of the epidemiology and pathogen distribution of bronchoalveolar lavage fluid in children with severe pulmonary infection: a retrospective study

**DOI:** 10.1186/s13052-025-01859-2

**Published:** 2025-01-28

**Authors:** Muchun Yu, Mingchao Li, Huiqing Sun

**Affiliations:** Department of Neonatology, Children’s Hospital Affiliated to Zhengzhou University, Henan Children’s Hospital, Zhengzhou Children’s Hospital, Henan, China

**Keywords:** Bronchoalveolar lavage fluid, Severe pulmonary infection, Etiology, Drug resistance

## Abstract

**Background:**

Severe pulmonary infection is the primary cause of death in children aged < 5 years. The early identification of pathogenic bacteria and targeted anti-infective therapies can significantly improve the prognosis of children with severe infections. This study aims to provide a reference for the rational use of antibiotics at an early stage in children with severe pulmonary infections.

**Methods:**

A retrospective, single-center longitudinal study included children with severe pulmonary infections between January 2017 and December 2022 by obtaining their bacterial culture results of bronchoalveolar lavage fluid.

**Results:**

This study included 4080 samples. The age of onset for severe pulmonary infection increased annually. The proportion of severe pulmonary infections across the different age groups and years was statistically significant (*p* < 0.001). Among children with severe pulmonary infections, bacilli were the most prevalent, followed by cocci and fungi. The predominant bacilli were *Acinetobacter baumannii* and *Klebsiella pneumoniae*. The predominant cocci identified in this study were *Streptococcus pneumoniae* and *Staphylococcus aureus*. The primary fungi included *Candida albicans* and *Aspergillus fumigatus*, which showed significant differences (*p* < 0.05). The incidence of drug-resistant bacteria has gradually declined, with infection rates of multidrug-resistant bacteria and extended-spectrum beta-lactamases consistently decreasing annually. For carbapenem-resistant *Acinetobacter baumannii* and *Pseudomonas aeruginosa*, the infection rates peaked in 2018, with statistical significance (*p* < 0.001).

**Conclusions:**

Severe pulmonary infections in children are significantly associated with age and types of infectious pathogens. Gram-negative bacteria are the primary cause of severe pulmonary infections in children. Clinicians should rationally use antibiotics according to the local distribution and drug resistance of pathogens, thereby enhancing therapeutic outcomes.

## Background

Severe pulmonary infection is a significant health concern among children worldwide and one of the leading causes of death in children aged < 5 years [1]. The development of severe pulmonary infection is affected by several factors such as etiology, autoimmunity, and complications, among which the etiology is of great importance [2–4]. Worldwide reports on the epidemiology and distribution of pathogens in severe pulmonary infections are often affected by age and region; however, there is still a lack of reliable reports in China [2, 5, 6]. Currently, the positive rate of clinical blood culture tests is low, and sputum culture tests are easily contaminated with oral bacteria, resulting in low specificity and accuracy of bacterial culture results. However, bronchoalveolar lavage fluid (BALF), which is collected using a bronchofibroscope in the pulmonary and sub-pulmonary segments, enhances the detection rate and accuracy of pathogens. This method has a significant practical value for the diagnosis and treatment of severe pulmonary infection [7]. In children with severe pulmonary infections, factors, such as genetic mutations of pathogens, inappropriate use of antibiotics, hormonal treatments, and invasive procedures contribute to a significant incidence of drug-resistant bacteria, leading to a decline in the efficacy of early clinical empirical anti-infective therapy [8, 9]. Therefore, early identification of pathogenic bacteria and implementing targeted anti-infection therapies can improve the prognosis of children with severe pulmonary infections [10]. This study aims to provide a reference for the rational use of antibiotics at an early stage in children with severe pulmonary infections.

## Methods

### Study design

A retrospective, single-center, longitudinal study was conducted in Children's Hospital Affiliated to Zhengzhou University between January 1, 2017 and December 31, 2022, focusing on the epidemic characteristics and drug resistance of the pathogens. Children with a definitive diagnosis of severe pulmonary infection and positive BALF culture results were included in this study. Clinical data for each child, including age at onset, clinical diagnosis, pathogenic bacteria cultured in BALF and drug resistance, were collected and analyzed. Children with positive BALF culture results were categorized into six groups according to the year (2017, 2018, 2019, 2020, 2021 and 2022) and the distribution and drug resistance of pathogens among these groups were compared. Additionally, the children were further divided into four age groups, namely, ≤ 28 days, 29 days—1 year, 1 year—3 years, and > 3 years, to assess potential differences in pathogen distribution in severe pulmonary infection across different age brackets.

### Participants

The study included children with severe pulmonary infections, aged 1 day after birth to 16 years, who required BALF analysis to identify pathogenic bacteria for anti-infection treatment. The exclusion criteria were as follows: (1) Congenital immune deficiency. (2) Congenital inherited metabolic diseases. (3) Congenital developmental malformation. (4) Patients with endocrine diseases or benign or malignant tumors. This study was approved by the Ethics Committee of Children’s Hospital Affiliated to Zhengzhou University.

### Specimen collection and detection

BALF samples from children with severe pulmonary infection in our hospital were collected between January 1, 2017, and December 31, 2022, excluding samples from children who had been hospitalized for an extended period (≥ 3 months). The results of the first bacterial isolation from the BALF were included in the study. BALF collection and cytological examinations were conducted in accordance with the “Chinese Expert Consensus on Cell Morphological Examination of Bronchoalveolar Lavage Fluid” [11]. Bronchoalveolar lavage was performed in accordance with the “Chinese Expert Consensus on Pathogen Detection of Bronchoalveolar Lavage in Pulmonary Infectious Diseases (2017 Edition)” [12]. BALF was collected by clinicians who strictly adhered to the aseptic protocols and expert consensus guidelines. The samples were sent to the bacterial culture laboratory by specialized nursing staff and processed within 30 min. The cultivation of bacteria or fungi was conducted following the fourth edition of the “National Clinical Laboratory Operating Rules” [13]. The treated samples were cultured at 35 °C with 5% CO_2_ in a Heal Force HF240 Automatic Culture Analyzer. Following pathogen identification, a drug sensitivity test was performed using the Automatic Microbial Analyzer/Drug Sensitivity Test System (VITEK 2-Compact Instrument). The minimum inhibitory concentration was determined using the dilution method and the results were interpreted according to the Clinical and Laboratory Standards Institute standards from 2012.

### Data analysis

Statistical analyses were conducted using SPSS version 25.0. Count data were expressed as rates (%), and comparisons between groups were performed using the χ^2^ test, the corrected χ^2^ test, and Fisher's exact test. Quantitative data were presented as the means ± standard deviation ($\overline{\chi }$ ± SD), and the rank sum test was used for group comparisons. Measurement data (nonnormally distributed) were expressed as medians, 25th percentiles and 75th percentiles [M (Q1, Q3)], and comparisons between groups were made using the Kruskal–Wallis test and analysis of variance. Differences were considered statistically significant at *p* < 0.05.

## Results

### Clinical baseline characteristics

During the study period from 2017 to 2022, 4080 children with positive alveolar lavage fluid cultures were identified, including 418 in 2017, 784 in 2018, 949 in 2019, 636 in 2020, 738 in 2021, and 555 in 2022. The onset age of severe pulmonary infection increased annually, with a maximum average onset age of 4.26 ± 3.96 years in 2021 and a minimum average onset age of 2.59 ± 2.56 years in 2017, indicating statistical significance (*p* < 0.001). From 2017 to 2022, the gender composition of children with severe pulmonary infection remained consistent, with a higher infection rate observed in males than in females. For children aged 0 to 12 months, the proportion decreased each year, with the highest rate being 181/418 (43.3%) in 2017 and the lowest being 108/555 (19.5%) in 2022, which was statistically significant (*p* < 0.001). Among children aged 1 to 3 years, the highest age ratio was 300/949 (31.6%) in 2019, whereas the lowest was 116/555 (20.9%) in 2022, which was also statistically significant (*p* < 0.001). As shown in Table 1.

**Table 1:** Clinical data of positive BALF culture in children from 2017 to 2022

	2017 (*n* = 418)	2018 (*n* = 784)	2019 (*n* = 949)	2020 (*n* = 636)	2021 (*n* = 738)	2022 (*n* = 555)	*χ*^*2*^	*P* value
Age *(* $\overline{\chi }$ ± SD)	2.59 ± 2.56	2.57 ± 2.66	2.91 ± 2.74	3.99 ± 3.70	4.26 ± 3.96	4.25 ± 3.58	16.082	< 0.001
Males, n (%)	239 (57.1)	484 (61.7)	575 (60.6)	373 (58.6)	466 (63.1)	312 (56.2)	9.344	0.096
0–12 months, n (%)	181 (43.3)	316 (40.3)	288 (30.3)	204 (32.1)	212 (28.7)	108 (19.5)	94.188	< 0.001
1–3 years, n (%)	90 (21.5)	238 (30.3)	300 (31.6)	169 (26.6)	190 (25.7)	116 (20.9)	32.163	< 0.001
> 3 years, n (%)	147 (35.2)	230 (29.3)	361 (38.0)	263 (41.3)	336 (45.5)	331 (59.6)	139.506	< 0.001

### Pathogenic distribution of bronchoalveolar lavage fluid

**Fig. 1:**
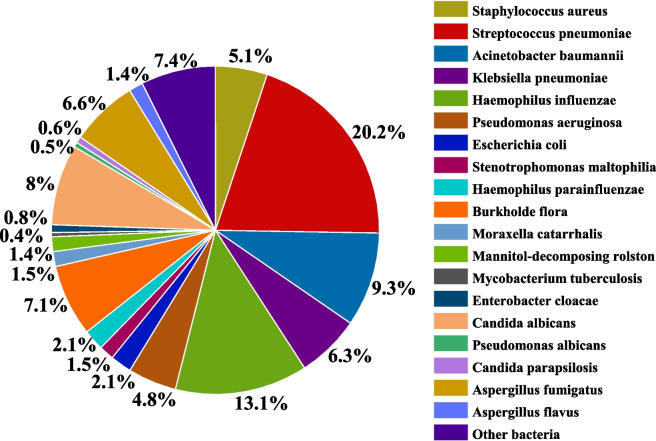
Proportion of pathogenic bacteria in BALF, 2017 to 2022

**Fig. 2:**
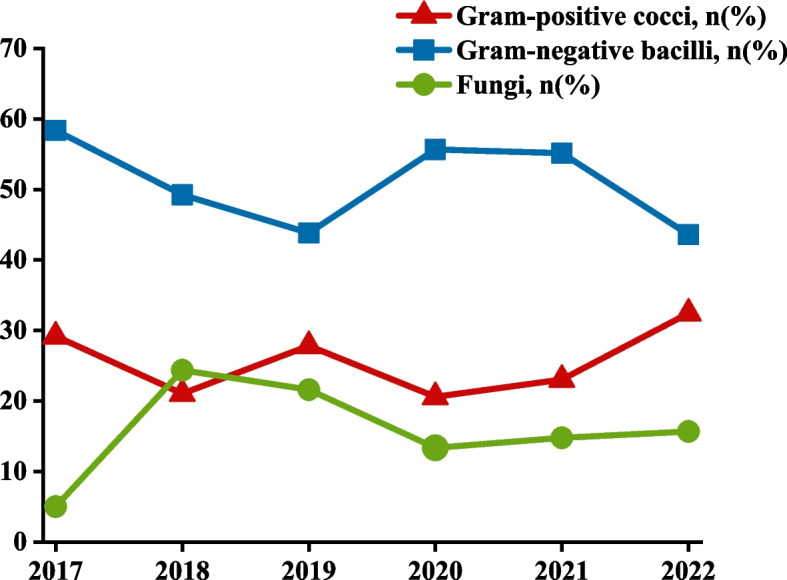
Dynamic changes of pathogenic bacteria in BALF, 2017 to 2022

In children with severe pulmonary infections and positive bacterial pathogens identified in BALF cultures, the proportion of bacilli was the highest, followed by cocci and fungi. The predominant bacilli were *Acinetobacter baumannii*, *Klebsiella pneumoniae*, *Haemophilus influenzae*, *Pseudomonas aeruginosa*, and *Burkholderia cepacia*. The primary cocci were *Streptococcus pneumoniae* and *Staphylococcus aureus*, whereas the main fungi were *Candida albicans* and *Aspergillus fumigatus*, as shown in Fig. 1. The bacilli infection rate decreased in 2019 and 2022, whereas that of cocci increased steadily. The infection rate of fungi was the highest in 2018 and 2019, followed by a decreasing trend from 2020 to 2023. As shown in Fig. 2.

*Streptococcus pneumoniae* was the dominant bacterium in gram-positive cocci infections, with higher infection rates recorded in 2017 and 2022 at 24.6% and 24.0%, respectively. The lowest infection rate was observed in 2020 at 15.3%, with statistical significance observed each year (*p* < 0.001). For *Staphylococcus aureus*, the highest infection rate was 8.5% in 2022, whereas the lowest was 3.4% in 2018, which was statistically significant across the years (*p* = 0.001). As shown in Table 2.

**Table 2:** Pathogenic distribution of BALF from 2017 to 2022, n (%)

	2017 (*n* = 418)	2018 (*n* = 784)	2019 (*n* = 949)	2020 (*n* = 636)	2021 (*n* = 738)	2022 (*n* = 555)	*χ*^*2*^	*P* value
**Gram-positive bacteria**								
*Staphylococcus aureus*	19 (4.5)	27 (3.4)	40 (4.2)	34 (5.3)	42 (5.7)	47 (8.5)	19.781	0.001
*Streptococcus pneumoniae*	103 (24.6)	138 (17.6)	224 (23.6)	97 (15.3)	128 (17.3)	133 (24.0)	33.524	< 0.001
**Gram-negative bacteria**								
*Acinetobacter baumannii*	67 (16.0)	112 (14.3)	63 (6.6)	58 (9.1)	39 (4.9)	41 (7.4)	69.954	< 0.001
*Klebsiella pneumoniae*	36 (8.6)	55 (7.0)	45 (4.7)	45 (7.1)	55 (7.5)	20 (3.6)	17.575	0.004
*Haemophilus influenzae*	49 (11.7)	80 (10.2)	168 (17.7)	47 (7.4)	119 (16.1)	71 (12.8)	48.363	< 0.001
*Pseudomonas aeruginosa*	33 (7.9)	24 (3.1)	26 (2.7)	47 (7.4)	39 (5.3)	27 (4.9)	32.458	< 0.001
*Escherichia coli*	18 (4.3)	22 (2.8)	17 (1.8)	10 (1.6)	13 (1.8)	6 (1.1)	16.252	0.006
*Stenotrophomonas maltophilia*	9 (2.2)	5 (0.6)	5 (0.5)	20 (3.1)	15 (2.0)	6 (1.1)	18.032	0.003
*Haemophilus parainfluenzae*	3 (0.7)	25 (3.2)	24 (2.5)	12 (1.9)	20 (2.7)	0	23.045	< 0.001
*Burkholde flora of onion*	13 (3.1)	47 (6.0)	38 (4.0)	72 (11.3)	75 (10.2)	44 (7.9)	53.693	< 0.001
*Moraxella catarrhalis*	14 (3.3)	3 (0.4)	0	13 (2.0)	10 (1.4)	20 (3.6)	49.689	< 0.001
*Mannitol-decomposing rolston bacteria*	0	13 (1.7)	18 (1.9)	17 (2.7)	7 (0.9)	4 (0.1)	17.786	0.003
*Mycobacterium tuberculosis*	2 (0.5)	3 (0.4)	4 (0.4)	7 (1.1)	0	1 (0.2)	11.067	0.050
*Enterobacter cloacae*	0	0	8 (0.8)	6 (0.9)	15 (2.0)	2 (0.4)	26.062	< 0.001
**Fungi**								
*Candida albicans*	4 (1.0)	41 (5.2)	93 (9.8)	54 (8.5)	74 (10.2)	59 (10.6)	50.262	< 0.001
*Pseudomonas albicans*	4 (1.0)	7 (0.9)	3 (0.3)	2 (0.3)	3 (0.4)	0	8.687	0.122
*Candida parapsilosis*	0	2 (0.3)	5 (0.5)	8 (1.3)	10 (1.4)	1 (0.2)	16.376	0.006
*Aspergillus fumigatus*	13 (3.1)	129 (16.5)	78 (8.2)	12 (1.9)	16 (2.1)	23 (4.1)	186.539	< 0.001
*Aspergillus flavus*	0	12 (1.5)	26 (2.7)	9 (1.4)	6 (0.8)	4 (0.7)	22.115	< 0.001
**Other bacteria**	31 (7.4)	42 (5.4)	64 (6.7)	66 (10.4)	52 (7.0)	46 (8.3)	14.41	0.013

*Acinetobacter baumannii*, *Haemophilus influenzae*, *Klebsiella pneumoniae*, and *Pseudomonas aeruginosa* were the predominant bacteria involved in gram-negative bacilli infections, with statistically significant infection rates observed each year (*p* < 0.05). The highest infection rate for *Acinetobacter baumannii* was 16.0% in 2017, and the lowest was 4.9% in 2021. For *Haemophilus influenzae*, the peak infection rate was 17.7% in 2019 and the lowest rate was 7.4% in 2020. The maximum infection rate of *Klebsiella pneumoniae* was 8.6% in 2017, with a minimum of 3.6% in 2022. The highest infection rate for *Pseudomonas aeruginosa* was 7.9% in 2017 and the lowest infection rate was 2.7% in 2019. The next most common bacteria included *Escherichia coli*, *Stenotrophomonas maltophilia*, and *Burkholderia cepacia*, with statistical significance in each year (*p* < 0.05). As shown in Table 2.

*Aspergillus fumigatus* was identified as the predominant pathogen in fungal infections, followed by *C. albicans*. The infection rate of *A. fumigatus* peaked at 16.5% in 2018 and reached a low level of 1.9% in 2020, with a statistical significance observed each year (*p* < 0.001). In contrast, the infection rate of *C. albicans* steadily increased over time, reaching a high value of 10.6% in 2022 and a low value of 1.0% in 2017, which was statistically significant for each year (*p* < 0.001), as shown in Table 2.

### Etiology comparison of bronchoalveolar lavage fluid at different ages

A comparison of the distribution of pathogenic bacteria across different age groups revealed significant differences in the infection rates of dominant bacteria, including gram-positive cocci, gram-negative bacilli, and fungal infections, with statistical significance observed in each group (*p* < 0.05). The infection rates of *Streptococcus pneumoniae* and *Staphylococcus aureus* were higher in children over 1 year old. Conversely, the infection rates of *Acinetobacter baumannii*, *Klebsiella pneumoniae*, *Escherichia coli*, *Burkholderia cepacia* complex, and *Mannitol-decomposing rolston bacteria* were elevated in children under 1 year old. Furthermore, for *Aspergillus fumigatus* and *Candida albicans* infections, the rates were also higher in children over 1 year old. As shown in Table 3.

**Table 3:** Etiological comparison of BALF in children, n (%)

	0–12 months (*n* = 1309)	1–3 years (*n* = 1103)	> 3 years (*n* = 1668)	*χ*^*2*^	*P* value
**Gram-positive bacteria**					
* Staphylococcus aureus*	45 (3.4)	46 (4.2)	117 (7.0)	22.081	< 0.001
* Streptococcus pneumoniae*	213 (16.3)	257 (23.3)	352 (21.1)	19.982	< 0.001
**Gram-negative bacteria**					
*Acinetobacter baumannii*	155 (11.8)	37 (3.4)	147 (8.8)	57.531	< 0.001
*Klebsiella pneumoniae*	164 (12.5)	39 (3.5)	51 (3.1)	131.426	< 0.001
*Haemophilus influenzae*	121 (9.2)	143 (13.0)	249 (14.9)	21.769	< 0.001
*Escherichia coli*	63 (4.8)	18 (1.6)	5 (0.3)	74.056	< 0.001
*Burkholde flora of onion*	115 (8.8)	73 (6.6)	103 (6.2)	8.148	0.017
*Pseudomonas aeruginosa*	32 (2.4)	35 (3.2)	123 (7.4)	47.635	< 0.001
*Stenotrophomonas maltophilia*	19 (1.5)	12 (1.1)	12 (0.7)	3.786	0.151
*Haemophilus parainfluenzae*	7 (0.5)	32 (2.9)	43 (2.6)	21.645	< 0.001
*Mannitol-decomposing rolston bacteria*	29 (2.2)	8 (0.7)	15 (0.9)	13.721	0.001
*Enterobacter cloacae*	16 (1.2)	14 (1.3)	4 (0.2)	12.043	0.002
*Mycobacterium tuberculosis*	2 (0.2)	2 (0.2)	10 (0.6)	5.438	0.066
**Fungi**					
*Candida albicans*	83 (6.3)	89 (8.1)	153 (9.2)	8.054	0.018
*Pseudomonas albicans*	11 (0.8)	12 (1.1)	5 (0.3)	6.723	0.035
*Candida parapsilosis*	16 (1.2)	2 (0.2)	8 (0.5)	11.352	0.003
*Aspergillus fumigatus*	68 (5.2)	85 (7.7)	118 (7.1)	6.939	0.031
*Aspergillus flavus*	7 (0.5)	25 (2.3)	25 (1.5)	13.244	0.001
**Other bacteria**	108 (8.3)	90 (8.2)	76 (4.6)	14.773	0.001

### Distribution of drug-resistant bacteria in bronchoalveolar lavage fluid

The incidence of drug-resistant bacteria gradually decreased, with the highest proportion recorded at 29.2% in 2017 and the lowest at 11.7% in 2022, indicating statistical significance (*p* < 0.001). The annual infection rates of CRE and MRSA were comparable, with no statistically significant differences. In contrast, MDRB and ESBLs infection rates exhibited a consistent downward trend over the years, with statistical significance (*p* < 0.001). CRABA and CRPAE infection rates peaked in 2018, whereas no infections were reported in 2017. As shown in Table 4.

**Table 4:** Distribution of drug-resistant bacteria in BALF from 2017 to 2022, n (%)

	2017 (*n* = 418)	2018 (*n* = 784)	2019 (*n* = 949)	2020 (*n* = 636)	2021 (*n* = 738)	2022 (*n* = 555)	*χ*^*2*^	*P* value
Incidence of drug-resistant bacteria (%)	122 (29.2)	184 (23.5)	124 (13.1)	141 (22.2)	109 (14.8)	65 (11.7)	93.323	< 0.001
CRE	22 (5.3)	28 (3.6)	37 (3.9)	36 (5.7)	34 (4.6)	17 (3.1)	7.464	0.188
MDRB	67 (16.0)	21 (4.3)	5 (0.5)	1 (0.2)	1 (0.1)	0	308.237	< 0.001
ESBLs	27 (6.5)	39 (5.0)	15 (1.6)	18 (2.8)	26 (3.5)	5 (0.9)	38.521	< 0.001
CRABA	0 (0)	72 (9.2)	50 (5.3)	42 (6.6)	18 (2.4)	20 (3.6)	67.553	< 0.001
CRPAE	0	11 (1.4)	3 (0.3)	25 (3.9)	7 (0.9)	6 (1.1)	32.453	< 0.001
MRSA	6 (1.4)	13 (1.7)	14 (1.5)	19 (3.0)	23 (3.1)	17 (3.1)	10.836	0.055

*CRE* Carbapenem-resistant Enterobacteriaceae, *MDRB* Multidrug-resistant Bacteria, *ESBLs* Extended Spectrum Beta-Lactamases, *CRABA* Carbapenem-resistant *Acinetobacter Baumannii*, *CRPAE* Carbapenem-resistant *Pseudomonas Aeruginosa*, *MRSA* Methicillin-resistant *Staphylococcus aureus*

## Discussion

Our study provided a comprehensive description of the epidemiology of severe pulmonary infections in children in central China. The six years of data in this study, including three years of the COVID-19 pandemic, showed that the age of onset and the distribution of pathogens in severe pulmonary infections vary from year to year. Our study provides a reference for rapid diagnosis of pathogens and early targeted anti-infection treatments. In addition, this study may provide a reference for pathogen changes during similar subsequent public health events.

Unlike other epidemiological studies, our study used BALF culture results for data analysis [2, 5, 6]. BALF is collected directly from lung lesions via bronchoscopy, and the detection rate of pathogens is higher in BALF than in sputum or blood [14]. Therefore, BALF culture is regarded as the gold standard for diagnosing pathogens in severe pulmonary infections and has been widely used for an extended period [15–18]. These findings indicate that BALF cultures can provide a reliable diagnostic basis in clinical practice.

Our study indicated that the positive rate of BALF culture in children with severe pulmonary infections at our hospital was notably high, with gram-negative bacteria identified as the predominant pathogens. This was similar to the results of a previous study conducted in China [18]. The principal gram-negative bacteria identified were *Haemophilus influenzae* and *Acinetobacter baumannii*. The main gram-positive cocci included *Streptococcus pneumoniae* and *Staphylococcus aureus*. The primary fungi detected in this study were *Candida albicans* and *Aspergillus fumigatus*. However, in a study in the USA, children with community-acquired pneumonia who required hospitalization were more likely to be infected with the virus or mycoplasma [19]. This may be due to the widespread use of conjugate vaccines against *Streptococcus pneumoniae* and *Haemophilus influenzae* type b [20, 21]. Antibiotics are widely used in China; however, the coverage rate of conjugate vaccines remains low [22]. Studies have suggested that increasing the coverage rate of conjugate vaccines can lead to a reduction in the colonization rate of pathogens in the upper respiratory tract and decrease in antibiotic resistance rates, which have good health benefits [22, 23]. Vaccination against bacterial pathogens, such as *Streptococcus pneumoniae* and *Haemophilus influenzae* type b, represents an effective strategy to reduce both morbidity and mortality associated with severe pulmonary infections in children [24].

Our study revealed that the proportion of children with severe pulmonary infections was higher in males than in females. In addition, there has been an annual increase in the age of children with severe pulmonary infections. Notably, the incidence of positive BALF cultures in infants aged 0 to 12 months showed a decreasing trend over the years, whereas the incidence in children aged > 3 years showed an increasing trend. In infants aged 0 to 12 months, gram-negative bacilli were the predominant pathogens associated with severe pulmonary infections. Conversely, in children over 1 year of age, the infection rates of gram-positive cocci, *Aspergillus fumigatus*, and *Candida albicans* were relatively high. The results may be related to the impact of the COVID-19 pandemic on pathogens since 2020 [25]. For example, in Li’ s study, the number and positivity rate of pathogens in the blood of neonates decreased during COVID-19, and the distribution of pathogens also changed [26]. This suggests that clinicians should fully consider the age and epidemic trends of children when empirically using antibiotics.

In our study, the incidence of drug-resistant bacteria gradually decreased, with the highest proportion of 29.2% in 2017 and the lowest of 11.7% in 2022, representing a significant reduction (*p* < 0.001). Except for CRE and MRSA, the infection rates of the MDR, ESBL, CRABA, and CRPAE strains exhibited a consistent downward trend annually (*p* < 0.001). This is similar to the results reported by the Infectious Disease Surveillance of Pediatrics program on bacterial epidemiology and antimicrobial resistance in children in China between 2016 and 2020 [27]. Drug-resistant bacteria are associated with an increased risk of treatment failure and relapse. Thus, they are important drivers of increased morbidity and mortality rates [28]. We inferred that this downward trend is attributable to the standardized use of broad-spectrum antibiotics in recent years and the COVID-19 pandemic [27, 29]. Bacterial adaptability and resistance can be minimized by standardizing and optimizing the use of antimicrobials, thereby reducing the emergence and spread of drug-resistant bacteria [29]. Improving the quality of antibiotic use is a primary objective of the World Health Organization's global action plan to combat antimicrobial resistance. Notably, there is substantial variation in the use of access, watch, and reserve antibiotics among neonates and children [30]. A report from the United Kingdom indicated that antibiotic use during primary health care significantly exceeded established guidelines, leading to excessive prescription [31]. Conversely, another report from Europe revealed that one-third of antibiotic prescriptions for children with fever were deemed unreasonable or uncertain, suggesting that antibiotic management should be implemented to limit usage until pathogenic bacteria are accurately identified [32]. Therefore, it is particularly important to analyze the distribution of drug resistance in severe pulmonary infections. This can guide clinicians in making empirical drug choices before determining the cause of the infection, thereby reducing inappropriate antibiotic use.

However, the COVID-19 pandemic has significantly increased the public awareness of infection prevention and hand hygiene. Parents paid more attention to their children's hygiene problems, such as washing hands and wearing masks. Restrictions on outdoor activities reduced the frequency of personal contact. These public health measures help to reduce the spread of pathogens and the risk of infection by drug-resistant bacteria [33]. Additionally, medical institutions have stepped up infection control measures, including hand hygiene, environmental cleaning, and equipment disinfection, which diminishes the likelihood of nosocomial transmission and cross-infection by drug-resistant bacteria [34].

The difference in pathogen distribution among hospitalized children with severe pulmonary infection was mainly reflected in the neonatal intensive care unit (NICU) and pediatric intensive care unit (PICU). Compared to the statistical results from the China Antimicrobial Resistance Surveillance System regarding the detection rates of pathogens in intensive care units across all age groups, notable differences were observed among *Klebsiella pneumoniae*, *Pseudomonas aeruginosa*, *Acinetobacter baumannii*, and *Streptococcus pneumoniae* [35]. The detection rate of drug-resistant bacteria, such as ESBLs and CREs, in children was higher than that in adult care units, which aligns with the infection characteristics observed in pediatric populations [36]. Therefore, it is essential to develop a nosocomial infection control strategy that specifically targets pathogenic bacteria associated with pediatric infections.

Our study had limitations. First, the data were obtained from a single tertiary hospital, which potentially limits the generalizability of our results to lower-level medical centers. Second, our study lacked a joint analysis of multi-center data, which may influence the scope and applicability of our findings. Finally, we did not analyze the specific antibiotic resistance of drug-resistant bacteria, hindering our ability to assess the appropriateness of antibiotic selection.

## Conclusions

Severe pulmonary infection in children is significantly associated with age and the type of infectious pathogen. Gram-negative bacteria are the primary cause of severe pulmonary infections in children. Vaccination, public health measures, personal protective measures, and the standardized use of antibiotics are of great significance in reducing pathogenic infections and the emergence of new drug-resistant strains. Clinicians should use antibiotics rationally and safely based on the local distribution of pathogens, pathogen culture results and drug sensitivity tests to optimize therapeutic outcomes.

## Data Availability

The datasets used and analyzed during the study are included in this published article.

## Abbreviations

BALF: Bronchoalveolar lavage fluid
CRABA: Carbapenem-resistant *Acinetobacter**baumannii*
CRE: Carbapenem-resistant Enterobacteriaceae
CRPAE: Carbapenem-resistant* Pseudomonas Aeruginosa*
ESBLs: Extended Spectrum Beta-Lactamases
MDRB: Multidrug-resistant bacteria
MRSA: Methicillin-resistant *Staphylococcus aureus*
NICU: Neonatal intensive care unit
PICU: Pediatric intensive care unit
